# Plasma polymeric immunoglobulin receptor exacerbates lung injury in *Klebsiella pneumoniae*-induced pneumosepsis

**DOI:** 10.3389/fimmu.2025.1624014

**Published:** 2025-06-26

**Authors:** Shuaiwei Wang, Hao Fu, Xiaoqing Li, Hongrui Xu, Yu Bai, Wenjun Jiang, Xiaozhe Cheng, Na Chen, Yijie Zhang, Wei Li

**Affiliations:** ^1^ Sepsis Laboratory, Center for Translational Medicine, The Second College of Clinical Medicine, Henan University, Kaifeng, Henan, China; ^2^ Department of Endocrine and Metabolic Diseases, The Fifth Affiliated Hospital of Zunyi Medical University, Zhuhai, Guangdong, China; ^3^ Clinical Laboratory, The First People’s Hospital, Shangqiu, Henan, China; ^4^ Department of Pulmonary and Critical Care Medicine, The Second College of Clinical Medicine, Henan University, Kaifeng, Henan, China; ^5^ Department of Clinical Medicine, Luohe Medical College, Luohe, Henan, China

**Keywords:** sepsis, polymeric immunoglobulin receptor, *Klebsiella pneumoniae*, alveolar type 2 epithelial cells, pyroptosis, caspase-11

## Abstract

**Background:**

Polymeric immunoglobulin receptors (pIgR) may enhance mucosal immunity or worsen an infection through transcytosis of polymeric immunoglobulins or infectious pathogens. The function of plasma pIgR in infections remains unknown.

**Methods:**

The association of plasma pIgR with the occurrence and prognosis of sepsis was investigated using human plasma. The role and underlying mechanisms of plasma pIgR were investigated in mouse models of sepsis and primary alveolar type 2 epithelial cells (AT2).

**Results:**

Quantitative proteomic and ELISA analysis revealed a significant association between plasma pIgR and the prognosis of patients of pneumonia-induced sepsis. Intravenous administrations of recombinant pIgR (r_pIgR) increased the mortality in mouse models of *Klebsiella pneumoniae* (KP)-induced pneumosepsis (KPS) and polymicrobial sepsis. r_pIgR also increased the injury score, caspase-11 and GSDMD-NT in the lungs of KPS mice. pIgR-neutralizing antibody (pIgR_Ab) exhibited opposite effects on animal survival in both sepsis models and on the injury score, caspase-11 and GSDMD-NT. Notably, r_pIgR did not affect the survival of Caspase-11-deficient KPS mice. pIgR immunoreactivity was absent in alveoli in normal mice, but emerged exclusively in AT2 in KPS mice. r_pIgR significantly reduced the level of biomarkers for AT2, but not AT1, whereas pIgR_Ab increased the level of AT2 biomarkers. In primary mouse AT2, heat-inactivated KP induced a marked increase in GSDMD-NT only in the presence of both r_pIgR and IgM.

**Conclusions:**

This study demonstrates that plasma pIgR is a potential prognostic marker for sepsis, and likely contributes to AT2 pyroptosis and sepsis lethality through interaction with IgM, indicating a broad pro-pathogenic role of plasma pIgR in infectious diseases.

## 1 Introduction

Polymeric immunoglobulin receptors (pIgR) are a type I glycosylated transmembrane protein, and are predominantly expressed on the basolateral surface of mucosal epithelial cells of the respiratory, digestive and urogenital tracts (1, 2). It is well-documented that pIgR bind to the “J” chain in polymeric immunoglobulins (pIgs), including dimeric IgA (dIgA) and polymeric IgM (IgM), and transport pIgs through transcytosis to the apical surface, where the N-terminal extracellular region of pIgR is hydrolyzed and released, together with the pIgs, into the mucus (1–3).

Previous reports of the function of pIgR in infectious diseases have been contradictory. On one hand, pIgR contribute to mucosal immunity by binding and killing infectious pathogens in the mucus (3–5). On the other hand, pIgR can facilitate the adherence and infection of host cells, such as epithelial and endothelial cells, by microbial pathogens including *Streptococcus pneumoniae*, *Candida albicans*, *Norovirus* and *Epstein-Barr virus* (6–10). dIgA, as one of the pIgR ligands, participates in both the protective and harmful activities of pIgR (3–10). In contrast, there has been no report on the involvement of IgM, the other ligand of pIgR, in the function of pIgR.

In addition to mucosal epithelium, pIgR are also found in the blood. The level of plasma pIgR is elevated under pathological conditions including biliary cholangitis, chronic obstructive pulmonary disease, malignant tumors and most recently acute respiratory distress syndrome (ARDS) (11–14). Previous studies have focused on pIgR in the mucus and pIgR-expressing cells, little is known about the functional significance of plasma pIgR in infectious diseases.

Sepsis is an infection-induced life-threatening organ dysfunction syndrome, and the leading cause of in-hospital mortality worldwide (15, 16). Among all infective diseases, infections in the lower respiratory tract (pneumonia) are associated not only with the most incidents, but also the highest mortality of sepsis (16–18). *Klebsiella pneumoniae* (KP) is one of the most common infective pathogens in sepsis-related pulmonary infections, and is associated with a mortality rate of 38% in sepsis patients (18).

Pyroptosis is a type of inflammatory cell death and plays a particularly important role in sepsis pathogenesis due to its association with the release of pro-inflammatory, damage-associated molecular patterns (19–22). Pyroptosis can be triggered by an activation of caspase-1 (Casp1)-mediated canonical inflammasome pathway or caspase-4/5/11 (Casp4/5 in human and Casp11 in mouse)-mediated non-canonical inflammasome pathway. In both pathways, activated caspases cleave gasdermin proteins, such as gasdermin D (GSDMD), into carboxyl- and amino-termini. The amino-termini (NT) then oligomerize, translocate, insert into plasma membrane and form highly permeative pores that lead to a rapid collapse of cross-membrane electrochemical gradients and cell death (20–22). Both Casp1- and Casp11-dependent pyroptosis are involved in tissue injury in animal models of sepsis (20, 21).

In our exploration of plasma proteins associated with sepsis lethality, quantitative proteomic analysis showed that the level of plasma pIgR in dying patients was significantly elevated. In this study, we investigated the association of plasma pIgR with the diagnosis, prognosis and pathogenesis of sepsis.

## 2 Materials and methods

### 2.1 Materials

Detailed information of reagents, including proteins, antibodies, animals, bacteria, chemicals and test kits, are summarized in **Supplementary Table 1**, unless otherwise specified.

### 2.2 Clinical study

The study involving human subjects was conducted in accordance with the Declaration of Helsinki, and the research protocol (No. 2016117) was approved by the Medical Ethical Committee of Henan University (23). Written informed consent was obtained prior to a subject’s enrollment into the study.

All human subjects were enrolled from departments in the Second College of Clinical Medicine of the Henan University, including 129 subjects with no existing medical conditions from the Center of Physical Examination (healthy group), 33 subjects of community-acquired pneumonia (CAP) from the Department of Pulmonary and Critical Care Medicine and 449 subjects of pneumonia-induced sepsis (PIS) from the Respiratory Intensive Care Unit (**Table 1**). CAP and sepsis were diagnosed according to respective guidelines (15, 24). PIS refers to sepsis patients that were initially diagnosed with pneumonia before developing into sepsis (SOFA score &≥ 2). The following exclusion criteria were applied in the enrollment of PIS subjects: pregnancy, recent chemotherapy, autoimmune diseases, HIV or HBV positive and recent surgery. There were no significant differences in the sex and age between healthy and CAP or between CAP and PIS groups. The mortality rate of PIS subjects was determined from hospital records or follow-up after discharge.

**Table 1:** Demography of sepsis patients.

Category	PIS
N	449
Sex, male/female	307/142
Age (median, IQR)	72, 62~80
Mechanic ventilation (%)	46.55
CAS/HAS	177/272
Septic shock (%)	26.45
28-day mortality (%)	39.66
Organ dysfunction (SOFA, mean ± SD)	
Sum	7.10 ± 3.83
Lung	2.76 ± 0.88
Heart	1.01 ± 1.68
Liver	0.38 ± 0.74
Kidney	0.69 ± 1.11
Central nervous system	1.44 ± 1.68
Coagulation	1.00 ± 1.18
Microbiology (%)^*^	
Gram-positive bacteria^#^	42.48
*Staphylococcus aureus*	15.04
*Corynebacterium*	8.27
*Enterococcus Faecium*	6.02
*Staphylococcus haemolyticus*	4.89
*Viridans Streptococci*	4.14
Gram-negative bacteria^#^	74.44
*Klebsiella pneumoniae*	39.47
*Acinetobacter baumannii*	36.84
*Pseudomonas aeruginosa*	9.02
*Escherichia coli*	5.64
*Stenotrophomonas maltophilia*	3.38
Fungus	36.84
*Candida albicans*	19.17
*Filamentous fungi*	4.51
*Candida krusei*	3.01
*Candida tropicalis*	1.88

PIS, pneumonia-induced sepsis; IQR, interquartile range; CAS, community-acquired sepsis; HAS, hospital-acquired sepsis; ^*^percentage of all positive sputum and blood cultures; ^#^5 most common pathogens.

### 2.3 Human blood collection and plasma preparation

One blood sample was obtained from each of the healthy and CAP subjects. For PIS subjects, blood samples were obtained on the day of sepsis diagnosis and at random times during subjects’ hospital stay (23). Plasma preparation was conducted as previously described (23). Briefly, blood samples were centrifuged at 2,000 g at 4 °C for 15 min within 2 h after collection, and the resultant plasma was aliquoted and then kept at -80 °C until use. Clinical test results corresponding to collected blood samples were obtained from hospital records.

### 2.4 Quantitative proteomic analysis

Twelve sepsis patients were selected from the PIS group based on the availability of both plasma samples collected when (1) the subject was in a stable condition according to SOFA scores (Moderate phase), and (2) the same subject was within 24 h from death due to condition deterioration (Moribund phase). Thus, a total of 12 pairs of plasma samples were selected, with each pair from one of the 12 patients at the moderate and moribund phases. The average interval between the moderate and moribund phase was 3.52 days.

Proteomic analysis of these plasma samples was performed using the tandem-mass tagged mass spectrometry (TMT-MS) at the MOE Key Laboratory of Bioinformatics, Tsinghua University (Beijing, China), as described previously (25). Briefly, 40 μL of each plasma samples was subjected to a depletion of abundant plasma proteins (IgY 14, Sigma, St. Louis, MO, USA), digestion with trypsin (Promega, Madison, USA), labeled with TMT (Thermo, Rockford, IL, USA), fractionated by a UPLC3000 system (Dionex, Sunnyvale, CA, USA), separated with an EASY-nLC 1000 system (Thermo Fisher Sci., Waltham, MA, USA) and a Q-Exactive HF-X spectrometer, and analyzed using Xcalibur 3.0.63 software (Thermo Fisher Sci., Waltham, MA, USA). The generated MS/MS spectra were searched against the Uniprot Human database (https://www.uniprot.org) using the SEQUEST searching engine in the Proteome Discoverer 2.1 software (PD, Thermo Fisher Sci., Waltham, MA, USA), which was also used for protein quantification according to the manufacturer’s instruction.

### 2.5 Animal models of sepsis

Nine- to ten-week-old male BALB/c, C57BL/6 or Casp11-knockout mice were used. All mice were housed with free access to food and water in a temperature and humidity-controlled animal room maintained on a 12–12 h light-dark cycle. The protocol for the animal study was approved by the Animal Care and Use Committee of the Medical School of the Henan University (Protocol No. HUSOM2020-113).

Animal anesthesia was induced and maintained by intramuscular injection(s) of ketamine hydrochloride (75 mg/kg) and xylazine (10 mg/kg). The mouse model of KP-induced pneumosepsis (KPS) was generated by an intratracheal administration of 0.5×10^9^ CFU KP in 40 μL sterile saline using a micro injector, as previously described (26). The mouse model of polymicrobial sepsis was induced by cecal ligation and puncture (CLP), as previously described (27). Briefly, the cecum was ligated at about 5 mm from the cecal tip, and then punctured with a 22-gauge needle to allow the leakage of cecal contents before placing the cecum back into the peritoneal cavity. The occurrence of animal death peaked between 24–48 h and 40–60 h after surgery in the CLP and KPS models, respectively, and largely stopped at 100 h in both models.

### 2.6 Measurement of pIgR in human and mouse plasma

The concentration of pIgR in human plasma was determined with a human pIgR ELISA kit (sensitivity range: 156.25–10,000 pg/ml) detecting the secreted form of human pIgR, according to the manufacturer’s protocol. The concentration of pIgR in plasma samples obtained on the day of sepsis diagnosis was used for subsequent analysis, unless otherwise specified.

The level of pIgR in mouse plasma was determined by Western blotting. Plasma from normal mice was used as control for KPS plasma that was obtained at 48 h post the induction of pneumosepsis.

### 2.7 Survival study

All animal experiments were conducted in a double-blind manner. Two strategies were used to determine the effect of plasma pIgR on sepsis lethality in both KPS and CLP mice, increasing the level of plasma pIgR by intravenous administrations of r_pIgR and reducing the biological activity of endogenous pIgR with a goat anti-mouse pIgR neutralizing antibody (pIgR_Ab). Saline and normal goat IgG were used as control for r_pIgR and pIgR_Ab, respectively. Mice were randomly divided into two groups immediately after surgery. Interventions were applied before the onset of mortality. For KPS mice, saline and r_pIgR (20 μg/kg) were injected at 18, 24 and 40 h after KPS induction. Goat IgG (100 μg/kg) and pIgR_Ab (100 μg/kg) were administered at 18 and 26 h post KPS induction. For CLP mice, r_pIgR, pIgR_Ab and their respective controls were injected at 20 and 28 h post CLP induction. Animal survival was monitored for 100 h or 7 days after sepsis induction. At the end of the observation period, animals were euthanized by CO_2_ inhalation.

For the assessment of tissue injury, KPS mice were euthanized at 48 h (saline and r_pIgR) or 40 h (goat IgG and pIgR_Ab) post KPS induction. A part of the lung was fixed with 4% paraformaldehyde for histological and immunohistochemical analysis, whereas the remaining lung tissue was used for Western blot analysis of biomarkers.

### 2.8 Lung histopathology

To determine pathological changes, KPS mouse lungs were fixed with 4% paraformaldehyde, embedded in paraffin, sectioned at 4 μm thickness and stained with hematoxylin and eosin to evaluate the degree of lung injury using a Pannoramic MIDI II system (3DHISTECH). Lung injury score was assessed according to the following parameters: alveolar capillary congestion, hemorrhage, inflammatory cell infiltration, alveolar wall thickness and hyaline membrane formation, as previously described (28). The percentage of lung injury was counted on a scale of 0–10: 0, not present (normal); 1–4, 10–40% (mild); 5–6, 50–60% (moderate); 7–8, 70–80% (severe); 9–10, 90–100% (very severe). The assessment of lung injury was conducted separately by two investigators in a double-blind manner. The lung injury scores reflect an average of assessed values.

### 2.9 Immunohistochemistry and immunofluorescence

The localization of pIgR in mouse lungs was examined using immunohistochemical and immunofluorescent staining. All immunostaining images were acquired with the Pannoramic MIDI II system, and analyzed with ImageJ and SlideViewer software.

Paraffin embedded lung sections were used for immunohistochemical staining of pIgR. Briefly, lung sections were treated with 0.2% hydrogen peroxide to block endogenous peroxidase, subjected to antigen retrieval, overnight incubation with the pIgR_Ab and 1-h incubation with HRP-conjugated secondary antibody before the reaction with 3,3’-diaminobenzidine. The sections were then counter-stained with hematoxylin.

Frozen sections were cut with a Cryostat (CM1950, Leica) and used for co-immunofluorescence staining of pIgR and cell markers, as previously described (27). Briefly, sections were blocked with 3% BSA and 0.1% Triton X-100 in PBS for 1 h at room temperature and incubated overnight at 4°C with different combinations of primary antibodies against pIgR, surfactant protein A (SPA) and C (SPC), Ly6G, CD68 and Casp11, which was followed by 1-h incubation at room temperature with corresponding Alexa Fluor 488-, Alexa Fluor 594- and DyLight550-conjugated secondary antibodies. Cell nuclei were stained with DAPI for 5 min at room temperature. Subsequently, cells immunolabelled with pIgR, Casp11, SPC and/or SPA antibodies were quantified using the ImageJ software.

### 2.10 Isolation and treatment of primary alveolar type 2 epithelial cells

The isolation and culture of AT2 were performed as previously described (29, 30). Briefly, lungs were removed from 4-week-old mice after euthanization, cut into 1 mm^2^ sizes in DMEM/F12 medium, digested sequentially in 0.25% trypsin and 0.1% collagenase I at 37°C, passed through a 200-mesh cell sieve and then centrifugated at 200g for 5 min. The resultant pellet was resuspended in complete culture medium and cultured in a humidified environment with 5% CO_2_ at 37°C temperature for 40 min. Nonadherent cells were collected, subjected to 3 rounds of centrifugation-culture, transferred to a rat IgG-coated culture dish for a 2-h incubation. AT2 were obtained after the lysis and removal of red blood cells from the nonadherent cells. The purity of AT2 was over 95%, as assessed by SPC immunofluorescent staining.

To determine the effect of pIgR on AT2 injury, primary AT2 were seeded in 6-well plates at 1×10^6^/mL per well. After 72 h, these cells were incubated with LPS (0.5 μg/mL), heat-inactivated KP (iKP, 2.5×10^7^ CFU), r_pIgR (0.5 μg/mL) and mouse IgM (0.5 μg/mL), alone or in combination, for 16 h before harvested for Western blotting analysis of pyroptosis markers, such as Casp11 and GSDMD-NT. Heat inactivation of KP was performed at 70°C for 30 min.

### 2.11 Western blotting

Mouse plasma, lung tissue and AT2 lysates were used for Western blotting, as previously described (27). For Western blotting of plasma pIgR, 0.25 μL plasma from each mouse was loaded in each lane. The protein concentration of the lung and AT2 lysates was determined using a BCA kit. Equal amount of protein from each sample was loaded for SDS-PAGE and subsequent Western blotting. Protein loading was further verified by Coomassie blue staining of the gel or Western blot of GAPDH. Transblotted membrane was blocked with 5% skim milk, incubated with one of the primary antibodies against pIgR, SPA, SPC, podoplanin (GP36), Casp11, Casp1, Casp3, Casp8 or Casp9, apoptosis-associated speck-like protein containing a CARD (ASC), GSDMD, receptor-interacting protein 3 (RIP3) and P62, and subsequently with a corresponding secondary HRP-conjugated antibody, before a final incubation in eECL solution. Chemiluminescent signal was acquired with a chemiluminescence imaging system (Amersham ImageQuant 800, Cytiva, Sweden). The relative intensity of protein bands was quantified using the ImageJ software.

### 2.12 Statistical analysis

All experiments involving animals and cultured cells were conducted 2–3 times. For *in vivo* studies, each treatment group contained 6–10 animals per experiment. For *in vitro* studies, each treatment contained duplicates or triplicates of samples. Data was analyzed using the IBM SPSS 25.0 and GraphPad Prism 8 software. The Shapiro-Wilk normality test was performed to determine the distribution of variables. The Student’s t-test was used for a comparison of two groups, or one-way ANOVA followed by Tukey test for multiple groups of Gaussian distributed variables. Non-Gaussian distributed data were compared using the Mann-Whitney U test for two groups, or the Kruskal-Wallis ANOVA test followed by the Dunn’s test for multiple groups. Survival curves were calculated using the Kaplan-Meier method and analyzed using the log-rank test; hazard ratio was estimated by Cox regression analysis. Variables were presented as mean ± standard deviation, unless otherwise specified. The statistical significance level was set at *p* < 0.05 after a two-tailed testing.

## 3 Results

### 3.1 Elevated pIgR level in plasma from moribund sepsis patients

**Figure 1:**
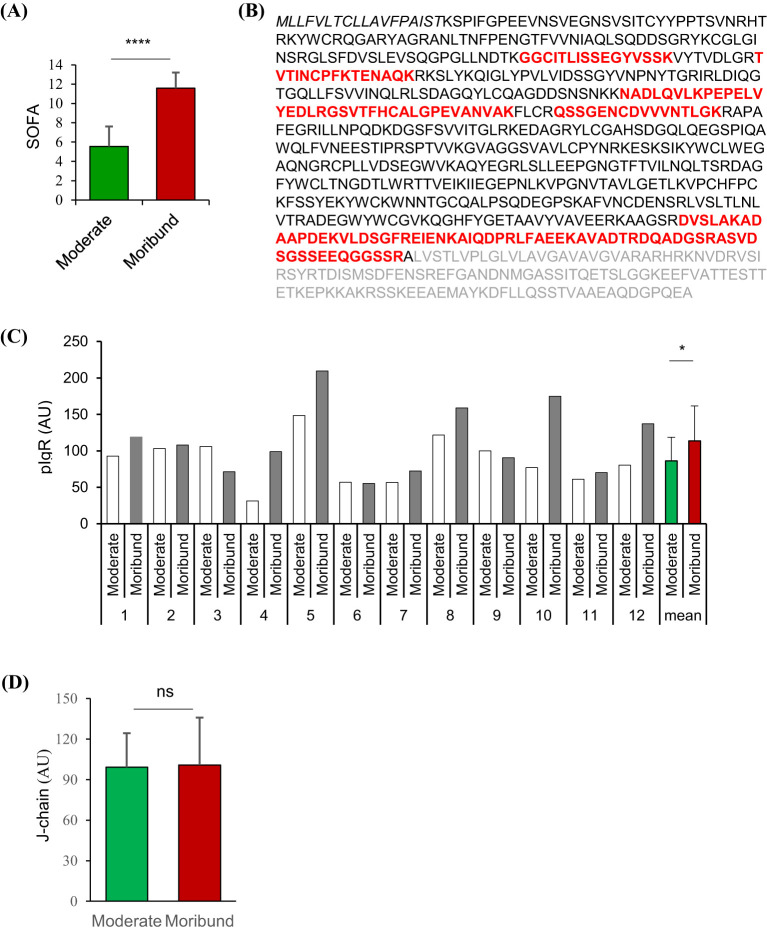
Quantitative proteomic analysis of polymeric immunoglobulin receptor (pIgR) and J-chain in paired plasma from sepsis patients. Tandem-mass tagged mass spectrometry was used to quantify proteins in paired plasma obtained from 12 sepsis patients when they were in moderate and moribund conditions. **(A)** Sepsis-related sequential organ failure assessment (SOFA) scores of sepsis patients when moderate and moribund plasma samples were obtained. **(B)** Amino acid sequence of the full-length human pIgR. Amino acids in bold letters are the sequence of the secreted pIgR. Unique pIgR peptides detected by mass spectrometry are shown in red. **(C)** Relative quantities of pIgR peptides in plasma obtained at moderate and moribund phases of the sepsis patients (1-12). **(D)** Relative quantities of J-chain peptides in respective plasma. ^ns^p > 0.05; ^*^p < 0.05; ^****^p < 0.0001. All *p* values were obtained by two-tailed paired t-test.

To screen plasma proteins associated with sepsis mortality, we selected 12 pairs of plasma samples from 12 PIS patients, each pair corresponding to the moderate (SOFA = 5.54 ± 0.60, **Figure 1A**) and moribund (SOFA = 11.58 ± 0.47, **Figure 1A**) phases of the same patient. Mass spectrometry detected 15 different pIgR-unique peptides, all of which are located in the extracellular domain of pIgR (red, **Figure 1B**), suggesting the presence of secreted pIgR in the plasma. Importantly, the quantity of pIgR peptides was 42.8% higher in the moribund than moderate plasma (*p* = 0.028, **Figure 1C**). In contrast to pIgR, the level of J-chain peptides, which reflects the level of pIgs, was not significantly different between moderate and moribund plasma (*p* = 0.875, **Figure 1D**), suggesting that the deterioration of sepsis is associated with increased pIgR, but not pIgs, in the blood.

### 3.2 Elevation of plasma pIgR is associated with sepsis mortality

To further characterize the association of plasma pIgR with sepsis. we determined pIgR concentrations in plasma from healthy (n = 129), CAP (n = 33) and PIS (n = 449) subjects. The demographic characteristics of PIS subjects are shown in **Table 1**. The SOFA score of PIS patients on the day of sepsis diagnosis was 7.10 ± 3.83. Approximately a quarter (26.45%) of PIS patients developed septic shock during hospitalization. The 28-day mortality rate was 39.66%. In agreement with other studies (17, 18), Gram-negative bacteria were more common than Gram-positive bacteria. Among all bacteria, KP was the most frequently identified in PIS subjects.

**Figure 2:**
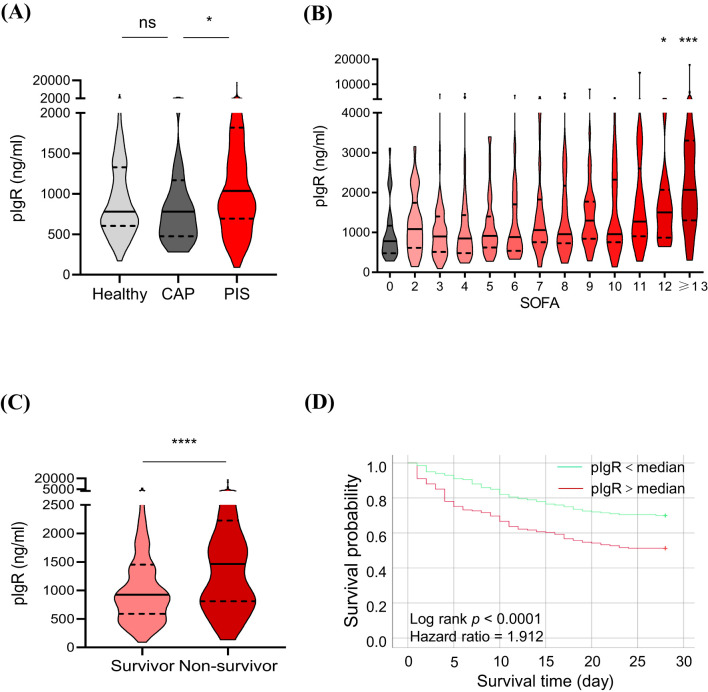
Association of plasma polymeric immunoglobulin receptor (pIgR) with the occurrence and prognosis of sepsis. **(A)** Comparisons of pIgR concentrations in plasma from healthy (n = 129), community-acquired pneumonia (CAP, n = 33) and pneumonia-induced sepsis (PIS, n = 449) subjects. Mann Whitney test was used to calculate p values. ^ns^p > 0.05; ^*^p < 0.05. **(B)** Comparisons of pIgR concentrations in CAP subjects (SOFA = 0, n = 33) with PIS subjects with different degrees of organ dysfunctions (SOFA scores). The n values are 29, 69, 48, 43, 40, 39, 25, 35, 33, 20, 22 and 46 for SOFA groups 2 to ≥ 13, respectively. Kruskal-Wallis test was used to calculate p values. *p < 0.05; ***p < 0.001. **(C)** A comparison of pIgR concentrations between PIS subjects that did (Survivor, n = 248) and did not (Non-survivor, n = 163) survive 28 days of hospitalization. ^*^p < 0.05; ^***^p < 0.001. **(D)** Log-rank (Mantel-Cox) test of Kaplan-Meier survival curves of PIS patients with a higher (> median, n = 225, red line) and those with a lower (< median, n = 224, green line) levels of plasma pIgR. ^****^p < 0.0001. Variables in **(A-C)** are presented as median ± interquartile ranges.

The median concentrations of pIgR were 779.76, 780.27 and 1041.87 ng/mL in healthy, CAP and PIS subjects, respectively (**Figure 2A**). pIgR concentrations were not different between healthy and CAP subjects (*p* = *0.47*), but 34% higher in PIS group (*p* = *0.01*, **Figure 2A**). Among PIS subjects, only those with multiple organ failures (SOFA score > 12) had a significantly higher level of pIgR than CAP subjects (SOFA = 0, **Figure 2B**). In addition, pIgR concentration was higher in PIS patients that were destined to die (1463 ng/ml) than those survived 28 days of hospitalization (924 ng/ml, *p < 0.0001*, **Figure 2C**). Consistent with the TMT-MS results, higher pIgR concentrations (> median) were associated with increased risk of death (hazard ratio = 1.912, *p < 0.0001*, **Figure 2D**).

### 3.3 Plasma pIgR contributes to sepsis lethality and lung injury

The association of plasma pIgR with sepsis mortality raised the possibility that the elevation of plasma pIgR may contribute to sepsis lethality. Given the prevalence of KP infection in PIS subjects, we decided to test the hypothesis in KPS mice, a model of KP-induced pneumosepsis.

**Figure 3:**
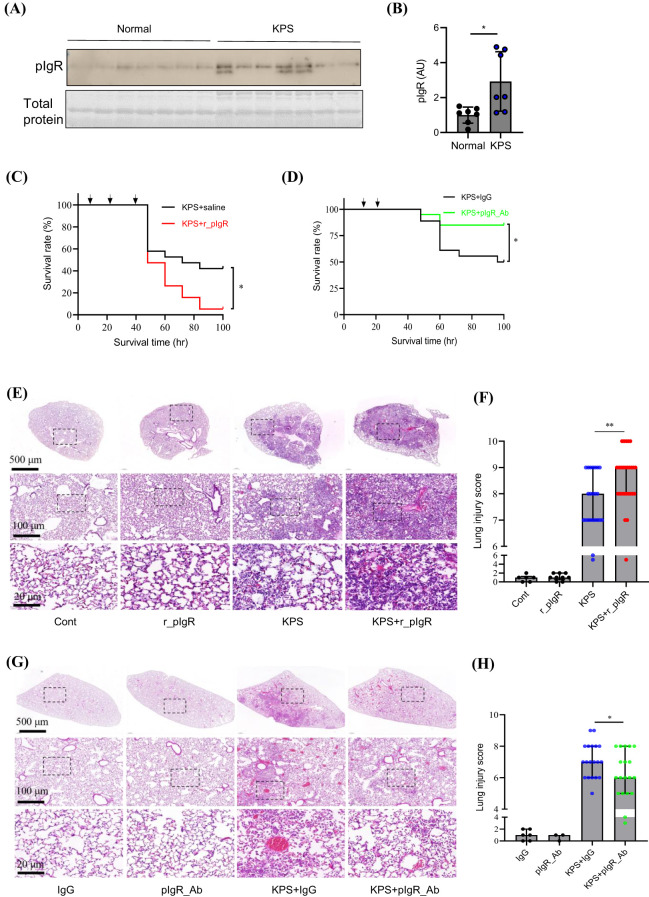
Effects of recombinant mouse polymeric immunoglobulin receptor (r_pIgR) and pIgR-neutralizing antibody (pIgR_Ab) on the survival and lung injury in mouse model of *K*. *pneumoniae* (KP)-induced pneumosepsis (KPS). KPS was induced by an intratracheal injection of KP (0.5 x 10^9^ CFU in 40 μL sterile saline). r_pIgR (20 μg/kg) or saline was injected thrice via tail veins at 18, 24 and 40 h after KPS induction. pIgR_Ab (100 μg/kg) or its control (100 μg/kg of goat IgG, IgG) was injected twice via tail veins at 18 and 26 h after KPS induction. **(A, B)** A comparison of pIgR concentrations in plasma from normal and KPS mice. KPS mouse plasma was obtained at 48 h post KP administration. **(C)** Effect of r_pIgR on the survival of KPS mice. n = 19. **(D)** Effect of pIgR_Ab on the survival of KPS mice. n = 18. **(E)** Representative images of H&E staining of normal (Cont) and KPS mouse lungs after indicated treatments. **(F)** Lung injury analysis based on H&E staining showing the effect of r_pIgR in KPS mice. n = 23 for KPS group, n = 27 for KPS+r_pIgR group. **(G)** Representative images of H&E staining of mouse lungs after indicated treatments. **(H)** Lung injury analysis showing the effect of pIgR_Ab in KPS mice. n = 19 for both KPS+IgG and KPS+pIgR_Ab groups. Arrows in **(C, D)** indicate the time of injections. p values reflect two-tailed Student’s t-test in **(B, H)**. Log-rank (Mantel-Cox) tests of Kaplan-Meier survival curves were performed in **(C, D)**. Mann-Whitney test was used in **(F)**. ^*^p < 0.05; ^**^p < 0.01.

Similar to PIS subjects, pIgR level was elevated by 193% in the plasma at 48 h after the induction of KPS (**Figures 3A, B**). To examine the role of increased plasma pIgR in sepsis, r_pIgR was administered intravenously at 20 μg/kg or 0.25 μg/ml blood in a mouse, which is approximately the range of elevation of pIgR concentration in sepsis patients. As shown in **Figure 3C**, r_pIgR reduced the survival rate of KPS mice from 42% to 5% (*p = 0.018*). In contrast to r_pIgR, pIgR-neutralizing antibody (pIgR_Ab) increased the survival rate from 50% to 85% (*p = 0.025*, **Figure 3D**). These results suggest that plasma pIgR contributes to the lethality of KP-induced pneumosepsis. Consistent with the effects of r_pIgR and pIgR_Ab on animal survival, histological analysis showed that administrations of r_pIgR and pIgR_Ab increased (**Figures 3E, F**) and decreased (**Figures 3G, H**) the lung injury score, respectively, demonstrating that plasma pIgR likely contributes to lung injury in KPS mice.

We next examined the effect of plasma pIgR on the survival of CLP mice, the most commonly used model of polymicrobial sepsis (**Supplementary Figure S1**). Similar to their effects in KPS mice, r_pIgR increased the mortality from 40% to 80% (*p = 0.084*), whereas pIgR_Ab reduced the mortality from 50% to 0% (*p = 0.025*). Together with observations in KPS mice, these results suggest that plasma pIgR contributes to the lethality of sepsis induced by infections at different organs.

### 3.4 Plasma pIgR accentuates AT2 injury

**Figure 4:**
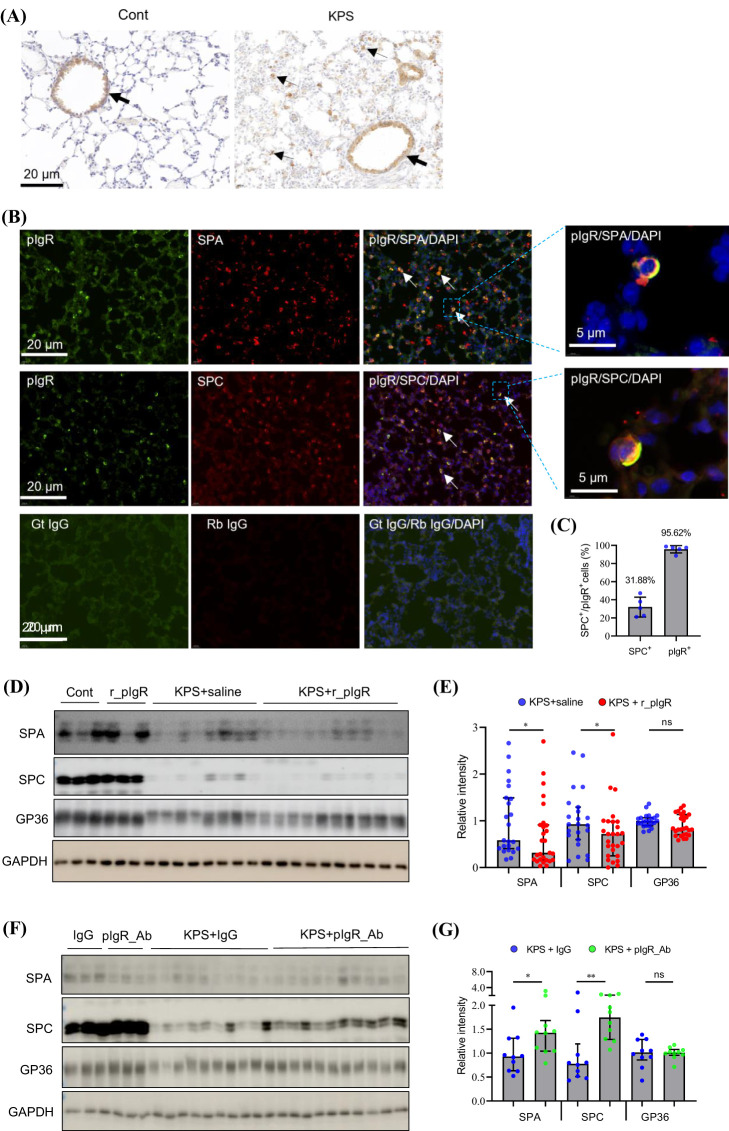
Association of plasma polymeric immunoglobulin receptor (pIgR) with the injury of alveolar type 2 epithelial cells (AT2) in mouse model of *K*. *pneumoniae* (KP)-induced pneumosepsis (KPS). **(A)** Immunohistochemical staining of pIgR in normal (Cont) and KPS mouse lungs (KPS). Thick arrows indicate pIgR staining in branchial epithelium. Thin arrows in KPS lungs indicate pIgR-positive alveolar cells. **(B)** Co-immunofluorescent staining of pIgR and AT2 cell markers, surfactant protein A (SPA, upper panel) and surfactant protein C (SPC, mid panel), in KPS mouse lungs. White arrows indicate cells co-stained with pIgR and SPA or SPC antibodies. The specificity of the immunostaining is shown in the bottom panel, in which the primary antibodies were replaced with respective goat (Gt) or rabbit (Rb) IgG. **(C)** The ratio of cells double-labelled with SPC and pIgR antibodies (SPC^+^/pIgR^+^) in all SPC (SPC^+^) or pIgR (pIgR^+^) positive cells. **(D, E)** Western blot analysis showing the effect of recombinant mouse pIgR (r_pIgR, i.v.) on the level of AT1 (GP36, also known as podoplanin) and AT2 (SPA and SPC) biomarkers in the lungs of mice subjected to indicated treatments. n = 23 in KPS group, n = 27 in KPS+r_pIgR group. **(F, G)** Western blot analysis showing the effect of pIgR-neutralizing antibody (pIgR_Ab, i.v.) on the level of SPA, SPC and GP36 in the lungs of mice subjected to indicated treatments. n = 10 in both KPS+IgG and KPS+pIgR_Ab groups. Variables in **(E, G)** are presented in median ± interquartile ranges, and *p* values reflect two-tailed Mann-Whitney tests. ^ns^p > 0.05; ^*^ *p* < 0.05; ^**^ *p* < 0.01.

To explore the mechanism of pIgR-induced lung injury, we first sought to determine pIgR-associated cells in the lung of KPS mice. Consistently with previous reports (11, 14), pIgR was only present in bronchial mucosal epithelial cells in normal lungs (thick arrows in **Figure 4A**, Cont). In KPS mice, intense pIgR staining was evident in numerous alveolar cells that resemble macrophages and AT2 (thin arrows in **Figure 4A**, KPS). Double immunofluorescence staining showed that pIgR-positive alveolar cells were mostly co-stained with SPA and SPC antibodies (arrows in **Figure 4B**). In particular, 96% of pIgR-stained alveolar cells were also positive for SPC (**Figure 4C**), suggesting a virtually exclusive localization of pIgR in AT2. Notably, pIgR immunofluorescence was often concentrated at the periphery of SPA or SPC staining (insets in **Figure 4B**), indicating the presence of pIgR on the plasma membrane of AT2. Unlike AT2, CD68-positive macrophages (**Supplementary Figure 2A**) and Ly6G-positive neutrophils (**Supplementary Figure 2B**) were essentially devoid of pIgR immunoreactivity (**Supplementary Figure 2**).

The induction of KPS caused a dramatic reduction in the level of biomarkers for AT1 (GP36) and AT2 (SPA and SPC) (**Figure 4D**). Administrations of r_pIgR resulted in significant decreases in SPA (33%, *p = 0.031*) and SPC (27%, *p = 0.046*), but not GP36 (*p = 0.125*, **Figures 4D, E**). Conversely, pIgR_Ab increased the levels of SPA (50%, *p = 0.023*) and SPC (73%, *p = 0.009*), with no impact on GP36 (**Figures 4F, G**). These observations are in agreement with the selective localization of pIgR in AT2, and indicate that AT2 are the primary target of plasma pIgR in KPS mouse lungs.

### 3.5 pIgR-induced lung injury is mediated by Casp11-dependent pyroptosis

**Figure 5:**
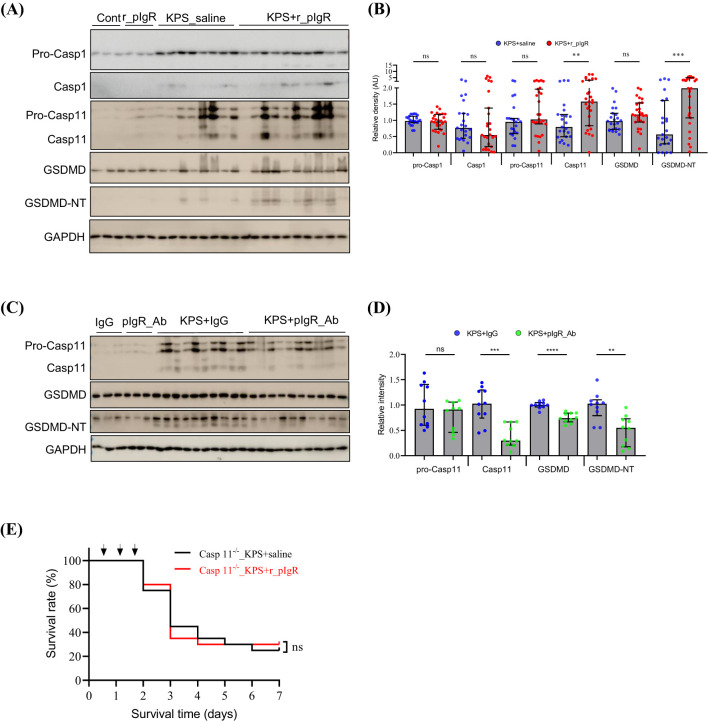
Association of plasma polymeric immunoglobulin receptor (pIgR) with caspase-11 (Casp11) and the N-terminal (NT) of gasdermin D (GSDMD) in the lungs of *K*. *pneumoniae* (KP)-infected (KPS) mice. **(A, B)** Western blot analysis of pro-caspase-1 (Pro-Casp1), Casp1, pro-Casp11, Casp11, GSDMD and GSDMD-NT showing the impact of recombinant mouse pIgR (r_pIgR, 20 μg/kg, i.v.). r_pIgR or saline (Cont) was administered thrice through tail veins in normal mice or in KPS mice at 18, 24 and 40 h after KPS induction. Mouse lungs were harvested at 48 h post KPS induction. n = 23 in KPS+saline group, n = 27 in KPS+r_pIgR group. Variables in **(B)** are presented as median ± interquartile ranges. p value reflects a two-tailed Mann-Whitney test. **(C, D)** Western blot analysis of pro-Casp11, Casp11, GSDMD and GSDMD-NT showing the impact of pIgR-neutralizing antibody (pIgR_Ab, 100 μg/kg, i.v.) in the lungs of KPS mice. Normal goat IgG (IgG, 100 μg/kg) was used as antibody control. n = 10 in both the KPS+IgG and KPS+pIgR_Ab groups. p value reflects a two-tailed Student’s t-test. **(E)** Effect of r_pIgR on the survival of Casp11-deficient KPS mice. n = 10, p value was determined by log-rank test of Kaplan-Meier survival curves. ^ns^p > 0.05; ^*^p < 0.05; ^**^p < 0.01; ^***^p < 0.001; ^****^p < 0.0001.

Sepsis-related cell injury may occur through a variety of mechanisms (19–22). Administrations of r_pIgR significantly increased the levels of Casp11 and GSDMD-NT by 99% and 254%, respectively, but not pro-Casp11, pro-Casp1, Casp1, pro-Casp8, Casp8, Casp9, ASC, RIP3 and p62 (**Figures 5A, B**; **Supplementary Figure 3**) in KPS mouse lungs. Consistent with the effects of r_pIgR, pIgR_Ab reduced the levels of Casp11, GSDMD and GSDMD-NT (**Figures 5C, D**). These observations indicate that plasma pIgR exacerbates lung injury in KPS mice by accentuating Casp11-dependent non-canonical pyroptosis. As expected, r_pIgR administrations did not have any effects on the survival of Casp11-deficient KPS mice (**Figure 5E**).

### 3.6 Both pIgR and IgM are required for the induction of AT2 pyroptosis by KP

We next examined whether pIgR can directly activate AT2 pyroptosis using cultured primary mouse AT2. As shown in **Supplementary Figure 4**, pIgR was not detected in normal and LPS-challenged primary mouse AT2, but present after r_pIgR treatment, suggesting that these cells do not express endogenous pIgR, but are capable of binding to extracellular pIgR. Notably, r_pIgR binding was significantly increased in LPS-challenged AT2 cells.

**Figure 6:**
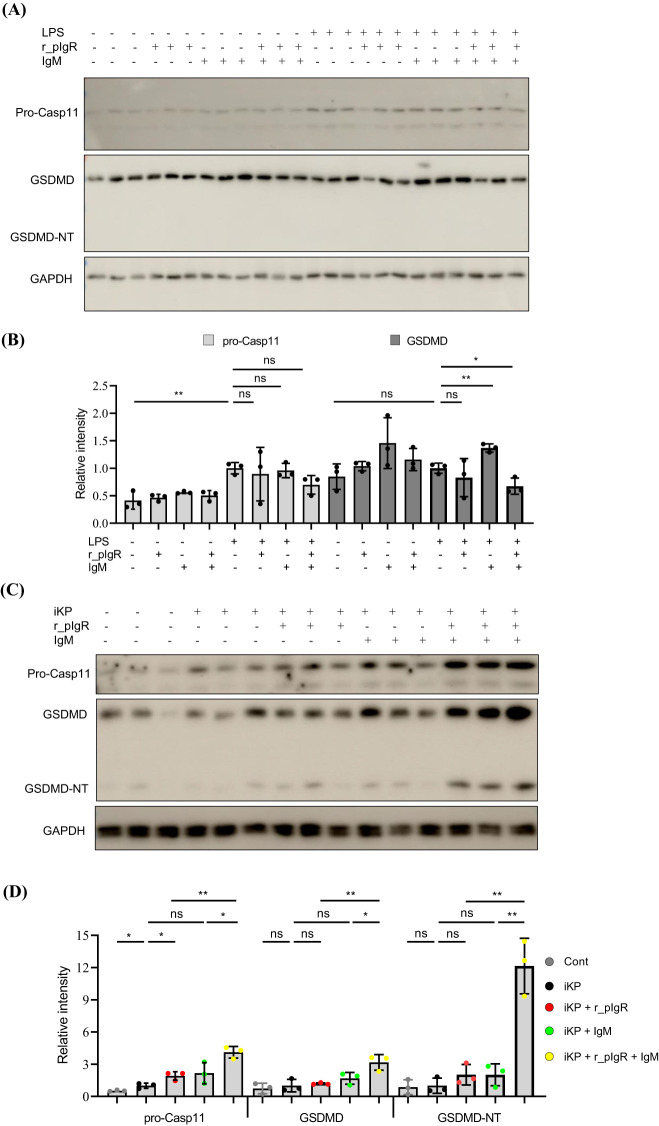
Activation of gasdermin D (GSDMD) in alveolar type 2 epithelial cells (AT2) by heat-inactivated *K. pneumoniae* (iKP), recombinant mouse polymeric immunoglobulin receptor (r_pIgR) and IgM. **(A, B)** Western blot analysis of pro-caspase-11 (pro-Casp11), GSDMD and the N-terminal of GSDMD (GSDMD-NT) in primary mouse AT2 treated with LPS (0.5 μg/mL), recombinant mouse pIgR (r_pIgR, 0.5 μg/mL) and/or IgM (0.5 μg/mL). **(C, D)** Western blot analysis of pro-Casp11, GSDMD and GSDMD-NT in primary mouse AT2 cells treated with iKP, r_pIgR (0.5 μg/mL) and/or IgM (0.5 μg/mL). n = 3; ^ns^p > 0.05; ^*^ *p* < 0.05; ^**^ *p* < 0.01. *p* values reflect two-tailed Student’s t-tests.

LPS treatment significantly increased the expression of pro-Casp11 (**Figures 6A, B**). However, a co-treatment of LPS with r_pIgR and IgM did not cause any further increase in the expression of pro-Casp11. Importantly, GSDMD-NT was not detected after any of these treatments, suggesting that r_pIgR, IgM and LPS, alone or in combination, were not sufficient in activating pyroptosis in AT2.

Similar to LPS, heat-inactivated KP (iKP) also increased the expression of pro-Casp11, but showed no significant effects on the levels of GSDMD and GSDMD-NT (**Figures 6C, D**). However, co-application of iKP, r_pIgR and IgM increased the levels of GSDMD by 326% (*p = 0.009*) and GSDMD-NT by a striking 13-fold, as compared with non-treated cells (**Figures 6C, D**), demonstrating that both pIgR and its ligand IgM are essential for the activation of AT2 pyroptosis by iKP.

## 4 Discussion

To date, there has been no report on the functional significance of plasma pIgR or pIgR-IgM immune complex. In this study, we characterized the association of plasma pIgR with the occurrence and prognosis of sepsis induced by pneumonia, and examined the role of plasma pIgR in the pathogenesis of pneumosepsis induced by pulmonary KP infection. The key findings include that the level of plasma pIgR is associated with the prognosis of PIS patients and that plasma pIgR aggravates the lethality of sepsis induced by pulmonary KP infection and peritoneal polymicrobial infection. In addition, the immune complex formed by pIgR and IgM contributes to the exacerbation of KP-induced non-canonical pyroptosis of AT2.

### 4.1 Elevation of plasma pIgR is an indicator of poor prognosis of pneumonia-induced sepsis

Earlier studies have showed that plasma pIgR was elevated in chronic sterile (or non-infectious) inflammatory diseases, including chronic obstructive pulmonary disease, biliary cholangitis and acute coronary syndrome (11, 12, 31). Little is known about the response of plasma pIgR to an infection until Gerard et al. reported recently an elevation of plasma pIgR in ARDS patients (14). However, the control group of this study were subjects with no respiratory disease and, therefore, it is not clear whether the elevation of plasma pIgR also occurs in subjects with non-ARDS, respiratory diseases. Nevertheless, the reported concentration of plasma pIgR is in line with our finding in PIS patients, which is not unexpected given that ARDS most commonly develops from pneumonia and non-pulmonary sepsis (32, 33). Comparisons of plasma from healthy, CAP and PIS subjects revealed a significant increase in pIgR in PIS, but not CAP subjects, suggesting that the occurrence of sepsis, not infection, is accompanied by elevated pIgR in the blood. However, plasma pIgR may not be a sensitive indicator for sepsis diagnosis since a significant elevation of plasma pIgR did not occur in PIS subjects unless the SOFA score reached 12 or higher, which represents a state of multiple organ failures.

On the other hand, the association of elevated plasma pIgR with multiple organ failures is in agreement with observations that pIgR level is higher in the moribund than moderate phase of sepsis, as shown by the TMT-MS analysis, and in non-survivors than survivors. Moreover, it is also consistent with the inverse correlation between the concentration of pIgR and survival probability. Together, these results point to a strong association of an elevation of plasma pIgR with a poorer prognosis of PIS patients.

### 4.2 A detrimental role of plasma pIgR in sepsis

Although there has been no report on the function of plasma pIgR, a harmful role of plasma pIgR was implied in several studies. For instance, a deficiency in pIgR expression resulted in reduced *S. pneumoniae* infection in the lung and brain (9, 10). Similar to the effect of pIgR deficiency, intravenous administration of the pIgR-neutralizing antibody also inhibited pneumococcal infection in the brain (10). Since *S. pneumoniae* was found to co-localize with pIgR immunoreactivity in endothelial cells in the blood-brain barrier, the effects of pIgR deficiency and pIgR_Ab were attributed to a blockade of pneumococcal binding to pIgR expressed by endothelial cells and a resultant inhibition of pneumococcal infection of these cells (10). However, pIgR deficiency eliminates not only pIgR expression in infected cells, but also the pIgR in the blood. Similarly, intravenously administered pIgR_Ab may bind and neutralize pIgR in the blood as well as in infected cells. Thus, it is possible that the observed inhibition of pneumococcal infection was a result from a depletion or inhibition of plasma pIgR. In current study, the effects of intravenous administrations of r_pIgR on the survival of both KPS and CLP mice provide a strong support of a detrimental role of plasma pIgR in sepsis.

The role of pIgR in infectious diseases is still a matter of debate in light of conflicting observations in previous studies (1–10). Global pIgR knockout has been an important and extensively used strategy in these studies (3–5, 9, 10), and resultant phenotypes cannot be readily attributable to a loss of plasma pIgR due to the elimination of local as well as circulating pIgR. Nevertheless, these studies raise the possibility that plasma pIgR is involved in the pathogenesis of infectious diseases caused by various pathogens. In addition to ARDS (14) and sepsis (this study), an elevation of plasma pIgR also occurs in several non-infectious (sterile) chronic inflammatory diseases (11–13, 31). It will be interesting to examine the role of plasma pIgR in these diseases. Findings from these future studies may potentially help resolve controversies over the function of pIgR.

### 4.3 AT2-targeting by plasma pIgR

KP infection causes widespread injuries of alveolar epithelial cells, such as AT1 and AT2, as indicated by the marked reduction in GP36, SPA and SPC in KPS mouse lungs. The significant impacts of r_pIgR and pIgR_Ab on SPA and SPC, but not GP36, suggest that AT2 are preferentially targeted by plasma pIgR. This notion is consistent with the virtually exclusive localization of pIgR immunoreactivity in AT2, among all alveolar cells, in KPS mouse lungs. Since AT2 do not express endogenous pIgR under physiological conditions or after bacterial infection (9, 11), the emergence of pIgR immunoreactivity likely results from a binding of extracellular pIgR. Indeed, pIgR was not detectable in primary AT2 cells before and after LPS treatment, but was evident after an exposure to r_pIgR. The further increase in pIgR in LPS-treated cells suggests that an infection by Gram-negative bacteria, such as KP, can augment the pIgR-binding capacity of AT2.

### 4.4 Accentuation of Casp11-dependent pyroptosis by plasma pIgR

Given that r_pIgR did not have a significant impact on Casp3, Casp8, Casp9, RIP3 and P62 in KPS mouse lungs, the injurious effect of plasma pIgR probably does not involve apoptosis, necroptosis or autophage-dependent cell death (19, 20). On the other hand, the opposing effects of r_pIgR (stimulatory) and pIgR_Ab (inhibitory) on GSDMD-NT suggest that pIgR exacerbates lung injury through accentuating cell pyroptosis. Notably, the level of biomarkers for Casp1-dependent canonical pathway, such as Casp1 and ASC, were not affected by r_pIgR or pIgR_Ab. In contrast, Casp11 was significantly and inversely impacted by r_pIgR and pIgR_Ab. It is thus reasonable to suggest that pIgR exacerbates lung injury primarily through augmenting Casp11-GSDMD-dependent non-canonical pyroptosis in KPS mice. This notion is supported by the observation that r_pIgR no longer affects the mortality of Casp11-deficient KPS mice.

Coincidentally, AT2 do not express Casp1, but are prone to Casp11-dependent pyroptosis during a bacterial infection (34, 35). The deficiency in Casp11 expression would result in a blockade of r_pIgR-induced AT2 pyroptosis. The loss of effects of r_pIgR on the mortality of Casp11-deficient KPS mice would thus suggest that the detrimental role of pIgR in KPS is probably attributable to its injurious effects on AT2.

### 4.5 pIgR and IgM in the activation of AT2 pyroptosis

One of the important observations in this study is that iKP, not LPS, can activate AT2 pyroptosis, as indicated by the level of GSDMD-NT. It has been controversial whether an extracellular application of LPS can cause AT2 injury. Several earlier studies showed that a 24-h treatment of human AT2 with LPS at 1 μg/mL reduced cell viability by 50% (36, 37). In other reports, however, human AT2 were found to be insensitive to extracellular LPS even when it was applied at extremely high concentrations (> 15 μg/mL) (38–40). Our results are in agreement with the later, suggesting that AT2 are not sensitive to LPS toxicity, which is probably due to the fact that LPS receptors, such as TLR2 and TLR4, reside in the cytoplasm of AT2 (35).

In contrast to LPS, infections by Gram-negative bacteria, such as *Burkholderia pseudomallei* and *Shigella flexneri*, can induce Casp11-dependent pyroptosis in human AT2 (41, 42). Interestingly, iKP alone failed to activate GSDMD in AT2. The reason for the discrepancy is not clear, but likely due to the loss of infectiousness of KP after heat-inactivation. In such case, iKP needs to be shuttled across the cell membrane to reach its receptor(s) in the cytoplasm (35). The requirement of r_pIgR and its ligand IgM for the activation of AT2 pyroptosis by iKP prompts us to propose that immune complexes formed by r_pIgR and IgM (**Supplementary Figure 5**) may serve as a carrier to shuttle iKP across the cell membrane.

There has been no report on the role of pIgR-IgM immune complexes in infectious diseases. pIgR, dIgA and IgM have each been shown to bind to different microorganisms, including *S. pneumoniae, Candida albicans* and *Neisseria meningitidis* (1, 7–9, 43). Immune complexes formed by pIgR and dIgA can bind to nasopharyngeal epithelial cells and trigger the internalization of *Epstein-Barr virus* or transcytosis of *S. pneumoniae* (7, 8). Thus, it might be reasonable to hypothesize that pIgR-IgM complexes bridge AT2 and iKP through direct binding, facilitate the internalization of iKP and lead to the activation of GSDMD-dependent pyroptosis. This hypothesis should be thoroughly tested in future *in vitro* and *in vivo* studies, which would help clarify molecular details of the mechanism underlying the detrimental role of plasma pIgR-IgM immune complex in AT2 injury and sepsis pathogenesis.

### 4.6 Limitations

Given that sepsis is most commonly developed from pneumonia (16–18), this study focused on the association of plasma pIgR with sepsis in PIS subjects. Future studies should determine the association of plasma pIgR with the occurrence and prognosis of sepsis caused by infections at other organs. In light of the prevalence of KP infection in sepsis patients (17, 18), KPS model was used to characterize the role of plasma pIgR in sepsis pathogenesis. Although the effects of r_pIgR and pIgR_Ab on the mortality of CLP mice suggest that plasma pIgR may also participate in the pathogenesis of sepsis caused by different infections, the underlying mechanisms remain to be elucidated. Moreover, characterizations of the role of plasma pIgR in sepsis were conducted using mouse sepsis models and primary mouse AT2 cells in this study. Observed effects of r_pIgR and pIgR_Ab should be confirmed using materials of human origin in the future., which help validify the usefulness of pIgR antibody-based strategies for the treatment of sepsis.

## 5 Conclusions

In this study, we find that plasma pIgR is a potential prognostic biomarker for pneumonia-induced sepsis and contributes to the lethality of sepsis caused by different infections. In addition, plasma pIgR aggravates lung injuries likely through an exacerbation of Casp11-dependent pyroptosis of AT2 in KP-induced pneumosepsis. pIgR-IgM immune complexes enable the activation of AT2 pyroptosis by KP. Together with previous studies (6–10), these findings raise the possibility that plasma pIgR, together with pIgs, plays a pro-pathogenic role in a broad range of infectious diseases. On the other hand, disrupting the interaction between plasma pIgR and pIgs may be a useful strategy for the development of sepsis therapy. In light of the protective effect of pIgR_Ab in both KPS and CLP sepsis models, pIgR-neutralizing antibodies targeting the binding site(s) of IgM and dIgA may have the potential for the treatment of sepsis that claims approximately 11 million human lives annually worldwide (16).

## Data Availability

The original contributions presented in the study are publicly available. This data can be found here: https://figshare.com/s/7b26ae5fa2d7a89c4161.
