# Correction: Plasma polymeric immunoglobulin receptor exacerbates lung injury in *Klebsiella pneumoniae*-induced pneumosepsis

**DOI:** 10.3389/fimmu.2025.1678276

**Published:** 2025-08-22

**Authors:** Shuaiwei Wang, Hao Fu, Xiaoqing Li, Hongrui Xu, Yu Bai, Wenjun Jiang, Xiaozhe Cheng, Na Chen, Yijie Zhang, Wei Li

**Affiliations:** ^1^ Sepsis Laboratory, Center for Translational Medicine, The Second College of Clinical Medicine, Henan University, Kaifeng, Henan, China; ^2^ Department of Endocrine and Metabolic Diseases, The Fifth Affiliated Hospital of Zunyi Medical University, Zhuhai, Guangdong, China; ^3^ Clinical Laboratory, The First People’s Hospital, Shangqiu, Henan, China; ^4^ Department of Pulmonary and Critical Care Medicine, The Second College of Clinical Medicine, Henan University, Kaifeng, Henan, China; ^5^ Department of Clinical Medicine, Luohe Medical College, Luohe, Henan, China

**Keywords:** sepsis, polymeric immunoglobulin receptor, *Klebsiella pneumoniae*, alveolar type 2 epithelial cells, pyroptosis, caspase-11

There was a mistake in the caption of **Figure 2B** as published The symbol (&) needs to be removed. The corrected caption of **Figure 2B** appears below.

**Figure 2:**
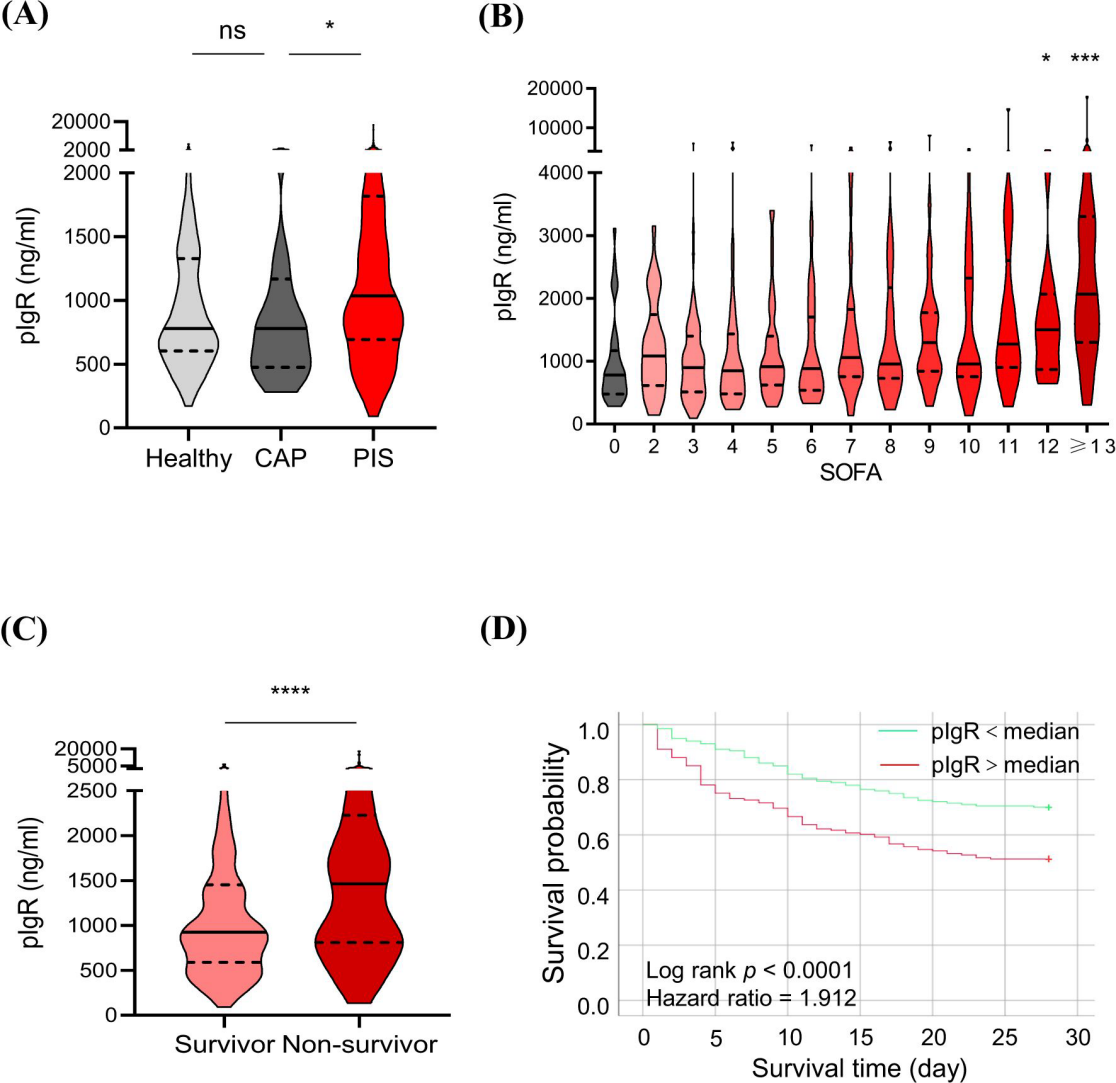
Association of plasma polymeric immunoglobulin receptor (pIgR) with the occurrence and prognosis of sepsis. **(A)** Comparisons of pIgR concentrations in plasma from healthy (n = 129), community-acquired pneumonia (CAP, n = 33) and pneumonia-induced sepsis (PIS, n = 449) subjects. Mann Whitney test was used to calculate p values. ^ns^p > 0.05; *p < 0.05. **(B)** Comparisons of pIgR concentrations in CAP subjects (SOFA = 0, n = 33) with PIS subjects with different degrees of organ dysfunctions (SOFA scores). The n values are 29, 69, 48, 43, 40, 39, 25, 35, 33, 20, 22 and 46 for SOFA groups 2 to ≥ 13, respectively. Kruskal-Wallis test was used to calculate p values. *p < 0.05; ***p < 0.001. **(C)** A comparison of pIgR concentrations between PIS subjects that did (Survivor, n = 248) and did not (Non-survivor, n = 163) survive 28 days of hospitalization. *p < 0.05; ***p < 0.001. **(D)** Log-rank (Mantel-Cox) test of Kaplan-Meier survival curves of PIS patients with a higher (> median, n = 225, red line) and those with a lower (< median, n = 224, green line) levels of plasma pIgR. ****p < 0.0001. Variables in **(A-C)** are presented as median ± interquartile ranges.

“(B) Comparisons of pIgR concentrations in CAP subjects (SOFA = 0, n = 33) with PIS subjects with different degrees of organ dysfunctions (SOFA scores). The n values are 29, 69, 48, 43, 40, 39, 25, 35, 33, 20, 22 and 46 for SOFA groups 2 to ≥ 13, respectively. Kruskal-Wallis test was used to calculate p values. *p < 0.05; ***p < 0.001”.

Also, there was an error in the article text, during the typographical process, an ampersand (&) was added to the body text of the article. A correction has been made to **3 Results**, *3.2 Elevation of plasma pIgR is associatedwith sepsis mortality*, Paragraph 3.

The corrected sentence appears below:

“The median concentrations of pIgR were 779.76, 780.27 and 1041.87 ng/mL in healthy, CAP and PIS subjects, respectively (**Figure 2A**). pIgR concentrations were not different between healthy and CAP subjects (*p = 0.47*), but 34% higher in PIS group (*p = 0.01*, **Figure 2A**). Among PIS subjects, only those with multiple organ failures (SOFA score > 12) had a significantly higher level of pIgR than CAP subjects (SOFA = 0, **Figure 2B**). In addition, pIgR concentration was higher in PIS patients that were destined to die (1463 ng/ml) than those survived 28 days of hospitalization (924 ng/ml, *p <0.0001*, **Figure 2C**). Consistent with the TMT-MS results, higher pIgR concentrations (> median) were associated with increased risk of death (hazard ratio = 1.912, *p < 0.0001*, **Figure 2D**)”.

Lastly, there was another error in the published article. The word ‘*not*’ is missing from the body text of the article. A correction has been made to **4 Discussion**, *4.3 AT2-targeting by plasma pIgR*, Paragraph 6.

“KP infection causes widespread injuries of alveolar epithelial cells, such as AT1 and AT2, as indicated by the marked reduction in GP36, SPA and SPC in KPS mouse lungs. The significant impacts of r_pIgR and pIgR_Ab on SPA and SPC, but not GP36, suggest that AT2 are preferentially targeted by plasma pIgR. This notion is consistent with the virtually exclusive localization of pIgR immunoreactivity in AT2, among all alveolar cells, in KPS mouse lungs. Since AT2 do not express endogenous pIgR under physiological conditions or after bacterial infection (9, 11), the emergence of pIgR immunoreactivity likely results from a binding of extracellular pIgR. Indeed, pIgR was not detectable in primary AT2 cells before and after LPS treatment, but was evident after an exposure to r_pIgR. The further increase in pIgR in LPS-treated cells suggests that an infection by Gram-negative bacteria, such as KP, can augment the pIgR-binding capacity of AT2”.

The original article has been updated.

