# Supplementary material for: Plasma polymeric immunoglobulin receptor exacerbates lung injury in Klebsiella pneumoniae-induced pneumosepsis
Source: Front Immunol. 2025 Jun 26;16:1624014. doi: 10.3389/fimmu.2025.1624014 (PMC12241074; doi:10.3389/fimmu.2025.1624014)
Supplement: Supplementary file 1 [file Table1.docx]

Supplementary Table 1. Reagent information

| Name | Cat. # | Supplier |
| --- | --- | --- |
| **Protein** | | |
| Recombinant mouse pIgR | 2800-PG | R&D Systems |
| Mouse IgM | C2115 | Applygen |
| Rat IgG | A7052 | Beyotime Biotechnology |
| Normal goat IgG | CR2 | Sino Biological |
| **ELISA** | | |
| Human pIgR | SEK10131 | Sino Biological |
| **Antibody** | | |
| Goat anti-pIgR IgG | AF2800 | R&D Systems |
| Rabbit anti-SPA IgG | 11850-1-AP | Proteintech |
| Rabbit anti-SPC IgG | 10774-1-AP | Proteintech |
| Goat anti-Podoplanin (GP36) IgG | AF3244 | R&D Systems |
| Rabbit anti-Ly6G IgG | GB11229 | Servicebio |
| Rabbit anti-CD68 IgG | GB113109 | Servicebio |
| Rabbit anti-caspase 1 IgG | ab179515 | Abcam |
| Rabbit anti-cleaved caspase 3 IgG | 9661S | Cell Signaling Technology |
| Rabbit anti-caspase 8 IgG | ab25901 | Abcam |
| Rabbit anti-caspase 9 IgG | 10380-1-AP | Proteintech |
| Rat anti-caspase 11 IgG | 14340s | Cell Signaling Technology |
| Rat anti-caspase 11 IgG | NB120-10454 | Novusbio |
| Rabbit anti-GSDMD IgG | HA721144 | HuaAn biotechnology |
| Rabbit anti-ASC IgG | GB113966 | Servicebio |
| Rabbit anti-RIP3 IgG | 95702s | Cell Signaling Technology |
| Rabbit anti-P62 IgG | ab91526 | Abcam |
| Rabbit anti-GAPDH IgG | 10494-1-AP | Proteintech |
| Rabbit anti-goat IgG-HRP | ZB-2306 | ZSGB-BIO |
| Donkey anti-rabbit IgG-HRP | NA934V | GE Healthcare |
| Donkey anti-goat IgG-HRP | SA00001-3 | Proteintech |
| Goat anti-rat IgG-HRP | SA00001-15 | Proteintech |
| Goat anti-mouse IgG-HRP | BA1050 | BOSTER |
| Donkey anti-rabbit IgG-Alexa Fluor 488 | A21206 | Thermo Fisher |
| Donkey anti-goat IgG-Alexa Fluor 488 | A11055 | Thermo Fisher |
| Donkey anti-rat IgG-DyLight 550 | SA5-10027 | Thermo Fisher |
| Donkey anti-rabbit IgG-Alexa Fluor 594 | A21207 | Thermo Fisher |
| Donkey anti-goat IgG-Alexa Fluor 594 | A11058 | Thermo Fisher |
| **Animals and Anesthetics** | | |
| BALB/c mice |  | SPF Biotechnology |
| Caspase 11^-/-^ mice |  | Xinxiang Medical University |
| S-ketamine hydrochloride |  | Hengrui Pharma |
| Xylazine |  | Aladdin |
| **Microbiology** | | |
| *Klebsiella pneumoniae* | ATCC 13883 | Fenghui Biotechnology |
| **Other chemical and biological reagents** | | |
| GelCode blue stain reagent | 24590 | Thermo Scientifc |
| LPS | L4130 | Sigma-Aldrich |
| Penicillin/Streptomycin | C3420-0100 | VivaCell BIOSCIENCES |
| DMEM/F12 | ZQ-600 | Zhong Qiao Xin Zhou Biotech. |
| Trypsin | C3530-0500 | VivaCell BIOSCIENCES |
| Fetal bovine serum | FBS-01UG-500 | Crystalgen |
| Collagenase I | C8140 | Solarbio |
| Red blood cell lysis solution | C3702 | Beyotime Biotech. |
| **Test kits** | | |
| BCA protein concentration determination kits | AR0146 | BOSTER |
| eECL Western blot kit | CW0049M | CWBIO |

**Supplementary Figure 1.** The impacts of plasma pIgR on sepsis lethality in a mouse model of polymicrobial sepsis induced by cecal ligation and puncture (CLP). **(A)** The effect of intravenous administrations (arrows) of recombinant pIgR (r_pIgR, 20 μg/kg/injection) on the survival rate of CLP mice (n = 10). Saline was used as the control for r_pIgR. **(B)** The effect of intravenous administrations (arrows) of a pIgR neutralizing antibody (pIgR_Ab, 100 μg/kg/injection) on the survival of CLP mice (n = 8). Normal goat IgG (IgG, 100 μg/kg/injection) was used as the control for pIgR_Ab. Kaplan-Meier survival curves were compared using the log-rank test. p values reflect two-tailed tests.


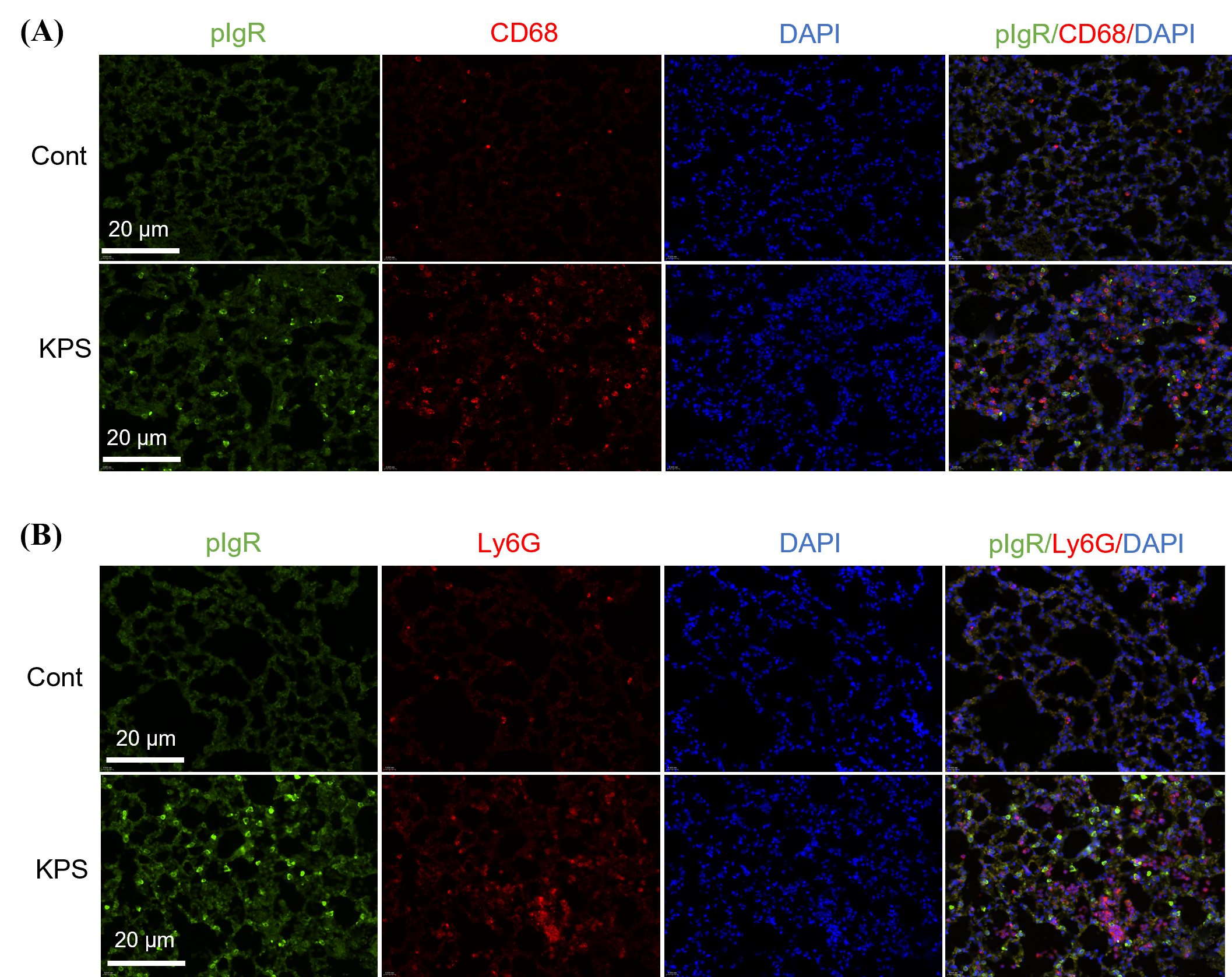


**Supplementary Figure 2.** Co-immunostaining of pIgR and macrophage marker CD68 or neutrophil marker Ly6G in mouse lungs. KPS was induced by intratracheal injection of *K. pneumoniae* (0.5 x 10^9^ CFU in 40 μL sterile saline). The lungs were fixed *in situ* with 4% paraformaldehyde at 48 h post KPS induction. **(A)** Representative images of pIgR (green) and CD68 (red) immunofluorescence in normal (Cont) and KPS mouse lungs. **B** Representative images of pIgR (green) and Ly6G (red) immunofluorescence in normal (Cont) and KPS mouse lungs.

**Supplementary Figure 3.** Western blots showing that intravenous administrations of recombinant pIgR (r_pIgR) had no significant effect on the levels of caspase 3 (Casp3), pro-caspase-8 (Pro-Casp8), Casp8, caspase-9 (Casp9), apoptosis-associated speck-like protein containing a CARD (ASC), receptor-interacting protein 3 (RIP3) and P62 in the lungs of KPS mice (**A, B)**. KPS was induced by intratracheal injection of K. pneumoniae (0.5 x 10^9^ CFU in 40 μL sterile saline). r_pIgR (20 μg/kg/injection) or its control (saline) was administered thrice at 18, 24 and 40 h after KPS induction. n = 6 in KPS + saline group, n = 7 in KPS + r_pIgR group; ns, *p* ＞0.05. p values reflect two-tailed Student’s t-tests.

**Supplementary Figure 4.** Expression and binding of polymeric immunoglobulin receptor (pIgR) by primary mouse alveolar type 2 epithelial cells (AT2). **(A)** AT2 cells were treated for 16 hr with LPS (0.5 μg/mL) and/or recombinant mouse pIgR (r_pIgR, 0.5 μg/mL), as indicated, before harvested for Western blot analysis of pIgR. **(B)** Relative intensity of pIgR protein band was quantified using ImageJ software. p value reflects a two-tailed Student’s t-test.

**Supplementary Figure 5.** Determination of IgM-r_pIgR binding by surface plasmon resonance. **(A)** Mouse IgM was immobilized on a CM5 sensor chip (Cytiva, Sweden) through amine coupling to reach a target density of 1500 response units. Recombinant mouse pIgR (r_pIgR) was injected at different concentrations (3.9, 15.625, 31.25, 62.5 and 125 nM) at a flow rate of 30 μL/min. The contact time was 90s, and the dissociation time was 2 min. **(B)** The sensorgram curve was generated and analyzed using Biacore T200 Evaluation Software 3.2.1., yielding a Kd of 68.54 nM.
